# OA11 Osteoporosis, denosumab, and hypocalcaemia in multimorbid renal impairment

**DOI:** 10.1093/rap/rkad070.011

**Published:** 2023-09-27

**Authors:** Jean-Luc Daurat, Spencer Ellis

**Affiliations:** Department of Rheumatology, Lister Hospital, East and North Hertfordshire NHS Trust, Stevenage, United Kingdom; Department of Rheumatology, Lister Hospital, East and North Hertfordshire NHS Trust, Stevenage, United Kingdom

## Abstract

**Introduction:**

Osteoporosis affects approximately 30% of patients with chronic kidney disease (CKD) at stages 3-5, with 10% of women and 5% of men experiencing a fragility fracture within a given three-year follow up period. First-line management of osteoporosis with bisphosphonates is often precluded in CKD, with alendronate and zoledronate being contraindicated in creatinine clearances of < 35mL/min, and risedronate if < 30mL/min. Denosumab, a RANK-ligand inhibiting monoclonal antibody, can be used in cases of poorer renal function, however causes hypocalcaemia more commonly than bisphosphonates. Concomitant CKD compounds this risk if creatinine clearance falls below 30mL/min, presenting additional challenges for management.

**Case description:**

A 75-year old woman with known osteoporosis and chronic kidney disease secondary to hypertension and a pauci-immune necrotising glomerulonephritis was found to have a serum calcium of 2.08mmol/L approximately three months after commencing six-monthly denosumab. Her lumbar spine T-score on dual x-ray absorptiometry showed severe osteoporosis at −2.9, with a fragility fracture of the humerus sustained four years prior. Her baseline eGFR was 23mL/min/1.73m2, and creatinine clearance was 22mL/min.

Her past medical history was extensive and included secondary hyperparathyroidism due to CKD and resulting vitamin D deficiency, with a PTH of 23.0pmol/L, serum calcium of 2.26mmol/L, and a serum 25-OH vitamin D3 level of 33.5nmol/L four months prior to denosumab therapy. The history also included Sjögren’s syndrome on long-term daily prednisolone 7.5mg, alternate-day hydroxychloroquine 200mg, and daily leflunomide 10mg for accompanying synovitis of the hands, hypothyroidism controlled with daily levothyroxine 50mcg, hypertension controlled with daily 2.5mg bisoprolol, peripheral neuropathy secondary to vitamin B12 deficiency on three-monthly hydroxocobalamin, myocardial infarction with percutaneous coronary intervention to three arteries, diverticular disease with abscess perforation requiring laparotomy and intensive care admission, and a previous deep vein thrombosis on long-term anticoagulation with apixaban.

The patient had received risedronate in the past while her creatinine clearance still permitted this. Her vitamin D deficiency had been managed orally with Adcal-D3 and colecalciferol 800IU once daily, which was uptitrated to 1600IU once daily prior to commencing denosumab. However, despite the patient’s serum vitamin D levels rising from 33.5 to 37.2nmol/L in the months preceding her first denosumab infusion, serum calcium had fallen below normal range on post-infusion blood tests. Serum phosphate returned at 0.91mmol/L, and vitamin D had fallen to 23.1nmol/L in the three months following denosumab treatment. Consequently, the patient was commenced on longer term intensive vitamin D supplementation.

**Discussion:**

This case raises the issue of multifactorial hypocalcaemia against a backdrop of osteoporosis, persistent vitamin D deficiency, and medical comorbidity limiting management options. The significance of CKD in this case highlights the importance of monitoring and replacing vitamin D. While the hypocalcaemia detected at three months post-infusion may have occurred in part due to the direct effect of denosumab, the patient’s vitamin D deficiency no doubt also played a role in this, and failing to detect the deficiency may have resulted in premature termination of denosumab treatment.

This case also highlights the extended timeframe in which such hypocalcaemia may present. Case-series literature describes hypocalcaemia manifesting at, on average, eight days post-infusion in patients with CKD-associated hyperparathyroidism. However we demonstrate here that extended lag times up to twelve weeks are possible; the patient was symptomatic with leg cramping at the time of clinic follow up.

Future practice may benefit from establishing monitoring regimes for those with CKD and osteoporosis receiving denosumab, or bisphosphonates toward the lower end of permitted renal function.

**Key learning points:**

Chronic kidney disease both limits available management options in osteoporosis, and increases the likelihood of complications from the remaining available agents – illustrated here with hypocalcaemia.

Hypocalcaemia in a patient with chronic kidney disease and osteoporosis may be driven by several factors: hyperphosphataemia, vitamin D deficiency, and the effect of denosumab to prevent mobilisation of calcium from bone to serum can all be culprits.

Calcium, phosphate, and vitamin D all need to be tightly monitored in medically complex patients on treatment for osteoporosis.

